# MiR-20a-5p Regulates MPP^+^-Induced Oxidative Stress and Neuroinflammation in HT22 Cells by Targeting IRF9/NF-*κ*B Axis

**DOI:** 10.1155/2021/6621206

**Published:** 2021-06-21

**Authors:** Qiang Wang, Yuan Wang, Feng Zhou, Jie Li, Gang Lu, Yingqian Zhao

**Affiliations:** ^1^College of Acu-Moxibustion and Massage, Shaanxi University of Chinese Medicine, Xianyang 712046, Shaanxi, China; ^2^The Affiliated Hospital of Shaanxi University of Chinese Medicine, Xianyang 712046, Shaanxi, China

## Abstract

Substantial evidence indicates that microRNAs (miRNAs) can be used as biological markers of Parkinson's disease (PD) and contribute to the risk assessment, early diagnosis, and treatment. We aimed to explore the role and potential mechanism of miR-20a-5p on inflammation and oxidative stress in 1-methyl-4-phenyl pyridine ion- (MPP^+^-) induced HT22 cells. HT22 cells were pretreated with miR-20a-5p mimic and/or pcDNA-IRF9 for 24 h and then treated with MPP^+^ (0.5 mM) for 24 h. The cell viability and apoptosis were determined using the Cell Counting Kit-8 (CCK-8) and Annexin V FITC/PI staining flow cytometry assay, respectively. The expression and secretion of inflammatory factors and oxidative stress-related factors were detected by enzyme-linked immunosorbent assay (ELISA). The protein expression levels were detected using Western blot analysis. Here, we discovered that MPP^+^ led to mitochondrial dysfunction, inflammation, and cell damage of HT22 cells, which were alleviated by miR-20a-5p overexpression. We further clarified that interferon regulatory factor 9 (IRF9) was a target gene of miR-20a-5p. IRF9 contributed to MPP^+^-induced mitochondrial disruption, inflammation, and cell apoptosis. Moreover, IRF9 hindered the improvement of miR-20a-5p overexpression on MPP^+^-induced neurotoxicity. Furthermore, the decrease of p-P65 level induced by miR-20a-5p mimic was significantly reversed by IRF9 overexpression. These findings demonstrate that miR-20a-5p contributes to MPP^+^-induced mitochondrial disruption and cell damage, and miR-20a-5p might be a novel therapeutic target for PD.

## 1. Introduction

Parkinson's disease (PD) is the second most common neurodegenerative disease of the extravertebral system after Alzheimer's disease (AD), which seriously threatens human health [1]. As the population ages, the incidence of PD is expected to rise sharply over the next 20 years [2]. The most important pathological change in PD is a progressive dopaminergic neuronal cell loss in the substantia nigra (SN) and development of Lewy bodies in dopaminergic neurons, resulting in extrapyramidal motor dysfunction, including tremor, rigidity, postural instability, and bradykinesia [3]. Studies have shown that the occurrence and development of PD are related to environmental toxins or the stress of the aging itself, which activate the chronic low-level inflammation in the brain [4, 5]. Subsequently, the gene mutation, oxidative stress, immunological abnormalities, and other mechanisms over time lead to the apoptosis, degeneration, and necrosis of dopaminergic nerve cells. At present, medical therapies (including pharmacotherapy and nonpharmacological approaches) and surgical therapies (such as deep brain stimulation) are the mainstays of treatment for PD. Notably, experimental therapies, including gene therapy, may be potentially utilized for diagnostic purposes and offer therapeutic targets to PD patients with an identified genetic cause(s) [6].

MicroRNAs (miRNAs) are endogenous single-stranded short sequence non-protein-encoded RNAs with a length of 19–23 nucleotides [7]. They can regulate the expression of functional genes by binding to target mRNAs and participate in the regulation of a variety of cellular processes, such as cell proliferation, differentiation, and apoptosis. Previous studies have shown that miRNA dysregulation leads to the onset of many diseases, including cancer and neurodegenerative diseases [8, 9]. Recently, growing studies showed that miRNAs participated in the development of PD, and some of the differentially expressed miRNAs, such as miR-7, miR-155, miR-124, and miR-22, were helpful for the diagnosis of PD [10–13]. Interestingly, previous bioinformatics analysis identified that miR-20a-5p expression was dramatically different between gray matter and white matter in AD brain [14]. Meanwhile, miR-20a-5p has been shown to be highly enriched in oligodendrocytes, and overexpression of miR-20a-5p decreased expression of the endogenous proteolipid protein (PLP) [15]. However, how miR-20a-5p is involved in the death of neurons remains unclear.

In the current study, we showed the protective effects of miR-20a-5p overexpression against 1-methyl-4-phenyl pyridine ion- (MPP^+^-) induced neurotoxicity in HT22 cells via targeting IRF9/NF-*κ*B axis.

## 2. Methods

### 2.1. Cell Culture and Treatment

The hippocampal cell line HT22 was purchased from the Procell Cell Bank (Wuhan, China). The cells were cultured in DMEM medium supplemented with 10% fetal bovine serum (FBS; Gibco-BRL Life Technologies, Paisley, UK) and 1% penicillin/streptomycin sulfate (Shzeye, Shanghai, China) at 37°C with 5% CO_2_ in a humidified incubator. To establish a model of PD in vitro, HT22 cells were treated with indicated concentration (0.25, 0.5, 1, and 2.5 mM) of MPP^+^ (Sigma, St. Louis, MO) for 24 hours. The sequence fragment of IRF9 was amplified by PCR and subcloned into pcDNA 3.1 vector (Invitrogen, Carlsbad, CA; pcDNA-IRF9) and sequenced. Lipofectamine 3000 transfection reagent was used for the transient transfection of NC mimic (Sangon Biotech, Shanghai, China), miR-20a-5p mimic (Sangon Biotech, Shanghai, China), and pcDNA-IRF9 according to the manufacturer's instructions.

### 2.2. Cell Survival Assay

Cell viability was assessed using the Cell Counting Kit-8 assay (CCK-8; Boster, Wuhan, China) according to the manufacturer's protocol. To be brief, the cells were digested with trypsin (7 × 10^4^/mL) and seeded in 96-well plate (1.0 × 10^3^ cells/well). Then, the seeded cells were incubated at 37°C and 5% CO_2_ for 48 hours. Finally, the supernatant was removed. 100 *μ*L CCK-8 solutions were added to each well and further incubated for 1 h at 37°C. The absorbance values were read at a wavelength of 450 nm.

### 2.3. Apoptosis Assay

The apoptosis of HT22 cells was assessed by Annexin V FITC/PI staining flow cytometry according to the manufacturer's instructions. The cells were washed with PBS (Invitrogen, Carlsbad, CA, USA) and adjusted the cell concentration to 7.0 × 10^4^ cells/mL. The cells were then resuspended with 500 *μ*L Binding Buffer, 5 *μ*L Annexin-V-FITC, and 5 *μ*L PI. The samples were protected from light in 4°C for 15 min and then analyzed by flow cytometry with excitation at 488 nm, and emission was measured at 560 nm.

### 2.4. Real-Time Fluorescence Quantitative Polymerase Chain Reaction (RT-qPCR)

Total RNA was isolated using TRIzol® reagent (Thermo Fisher, Massachusetts, USA). SYBR Premix Ex Taq kit (Bao Biological Engineering, Dalian, China) was used following the guidelines. The reverse transcriptional reaction condition was as follows: 95°C for 30 s, 40 cycles of 95°C for 5 s, and 60°C for 30 s. Sequences of primers used in this study were as follows: miR-20a-5p-forward, 5'-UAA AGU GCU UAU AGU GCA GGU AG-3', miR-20a-5p-reverse, 5'-CUA CCU GCA CUA UAA GCA CUU UA-3'. The relative gene expression level was determined using the 2^−△△Ct^ method on ABI software, Foster City, CA.

### 2.5. Dual-Luciferase Assay

The wild-type pSI-Check2-m-IRF9 (IRF9-3'UTR-wt) and the mutant pSI-Check2-m-IRF9 (IRF9-3'UTR-mut) recombinant dual-luciferase reporter plasmid were designed and synthesized based on the binding region of miR-20a-5p and IRF9 3'UTR sequence. The 293T cells were cotransfected with miR-20a-5p mimic/NC mimic and IRF9-3'UTR-wt/IRF9-3'UTR-mut by Lipofectamine™ 3000 (Invitrogen). After 48 hours, the luciferase activities were analyzed using Promega Dual-Luciferase system (Promega, Madison, Wisconsin, WI, USA).

### 2.6. Enzyme-Linked Immunosorbent Assay (ELISA)

The contents of IL-1*β*, IL-6, and TNF-*α* in the HT22 cells and supernatant were assayed using corresponding ELISA kits according to the manufacturer's protocol (Aci BIO, Shanghai, China).

### 2.7. Western Blot Analysis

Total protein from HT22 cells was lysed with RIPA lysis buffer (P0013B; Beyotime, Jiangsu, China) containing 1% PMSF following the manufacturer's protocol. The protein concentration of each sample was measured using a BCA Protein Quantification Kit (Westang, Shanghai, China). Total protein samples were standardized and electrophoresed on 10% SDS-PAGE gel and then transferred to nitrocellulose membranes (Hybond, USA). The membranes were blocked with 5% nonfat milk, then washed in triplicate with TBST, and incubated at 4°C overnight with primary antibodies. Then, the membranes were washed in triplicate with TBST and incubated with HRP Goat Anti-Rabbit IgG (Abcam, Cambridge, UK; cat. no. ab6721) at a dilution of 1 : 10000 for 2 h. The membranes were washed in triplicate with TBST. Membrane enhanced chemiluminescence (ECL) detection reagent (Reagent A : Reagent B = 1 : 1) reaction was performed for 2 min, the film was removed, the excess liquid was shaken off, PVDF film was wrapped with a plastic wrap, and X film was kept in the dark room for sensitization, development, and fixing. The net optical density was analyzed with the gel Image processing system (Image-pro Plus 6.0). Primary antibodies used were as follows: Bax (Abcam, Cambridge, UK; cat. no. ab32503), Bcl-2 (Abcam, Cambridge, UK; cat. no. ab32124), Caspase 3 (Abcam, Cambridge, UK; cat. no. ab13847), Cleaved-Caspase 3 (Abcam, Cambridge, UK; cat. no. ab2302), cytochrome-c (Affinity Biosciences, OH, USA; cat. no. AF0146), AIF (Abcam, Cambridge, UK; cat. no. ab1998), IRF9 (Abcam, Cambridge, UK; cat. no. ab126940), and *β*-actin (Abcam, Cambridge, UK; cat. no. ab8227).

### 2.8. ROS Detection

The changes of ROS level in HT22 cells were measured using 2',7'-dichlorofluorescein-diacetate (DCFH-DA, Sigma-Aldrich, MO, USA) staining according to the manufacturer's protocol (Beyotime, Shanghai, China). The cells were seeded into 6-well plate (1.0 × 10^6^ cells per well) and stained with 10 *μ*mol/L DCFH-DA for 30 min at 37°C in the dark. The ROS generation was analyzed by the flow cytometer (Bender MedSystems, CA, USA) and CytExpert software.

### 2.9. Mitochondrial Membrane Potential (MMP) Measurement

MMP of HT22 cells was tested using fluorescent probe JC-1 (Nanjing KeyGen Biotech. Co. Ltd., Jiangsu, China). The cells were incubated with JC-1 (10 *μ*M) at 37°C for 30 min. Afterwards, the cells were rinsed and suspended with 1× incubation buffer. The level of MMP was detected by the flow cytometer (Bender MedSystems, CA, USA) and CytExpert software.

### 2.10. Statistical Analysis

Statistical analysis was performed using SPSS 19.0 (IBM Corp., Armonk, NY, USA). The data are expressed as the mean ± standard deviation. All experiments were repeated six times. Differences among multiple groups were compared by one-way analysis of variance (ANOVA) with Dunnett's post hoc test, and differences between two groups were compared by Dunnett's *t*-test. *P* < 0.05 was considered statistically significant, and <0.01 was considered highly significant.

## 3. Results

### 3.1. MiR-20a-5p Levels Were Decreased in MPP^+^-Treated HT22 Cells

**Figure 1:**
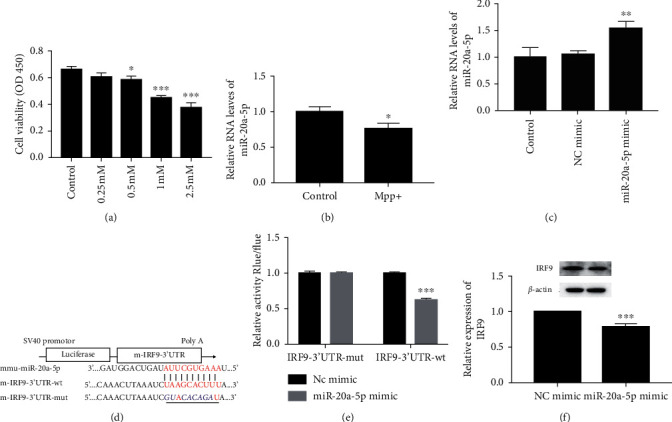
MiR-20a-5p level was decreased in MPP^+^-treated HT22 cells. (1) The cell viability was determined using CCK-8-solution for the cells treated by different concentration of MPP^+^ (0.25, 0.5, 1.0, and 2.5 mM). (b and c) The level of miR-20a-5p was tested by RT-qPCR assay.  ^*∗*^*P* < 0.05 vs. control,  ^∗∗^*P* < 0.01 vs. control, and  ^∗∗∗^*P* < 0.001 vs. control. (d) Sequence alignment of miR-20a-5p and the 3'-UTR of IRF9. (e) MiR-20a-5p mimic suppressed the expression of 3'-UTR-luciferase reporter of IRF9 in 293T cells, but the mutant vector was immune to miR-20a-5p. (f) The protein level of IRF9 was tested by Western blot analysis. *β*-actin is a loading control.  ^∗∗∗^*P* < 0.001 vs. NC mimic. Data are expressed as mean ± SD. The experiments were repeated six times.

In this study, we determined that the optimal induction concentration of MPP^+^ was 0.5 mM, at which the cell viability of HT22 cells was decreased to about 55% (Figure 1(a)). To explore whether miR-20a-5p is involved in MPP^+^-induced cell damage in HT22 cells, we firstly tested the level of miR-20a-5p in MPP^+^-induced HT22 cells. We found that miR-20a-5p level was significantly decreased compared with the control group when the cells were treated with 0.5 mM MPP^+^ (Figure 1(b)). These changes indicated that miR-20a-5p might contribute to the cell damage induced by MPP^+^.

### 3.2. IRF9 Is a Target Gene of MiR-20a-5p

In order to investigate the effect and the potential molecular mechanism of miR-20a-5p on MPP^+^-induced HT22 cell injury, we firstly designed and synthesized an effective miR-20a-5p mimic (Figure 1(c)). Bioinformatics prediction software shows that the mRNA of miR-20a-5p has the binding site of IRF9 3'UTR (Figure 1(d)). Meanwhile, the results of double fluorescence reporter gene system showed that the luciferase activity of the IRF9 3'UTR-wt in the miR-20a-5p mimic group was significantly lower than that in the NC group (Figure 1(e)). Western blot analysis proved that the protein level of IRF9 was decreased in miR-20a-5p mimic-transfected HT22 cells (Figure 1(f)). Thus, miR-20a-5p could target mRNA of IRF9 and negatively regulated its expression.

### 3.3. IRF9 Reversed Cell Apoptosis Inhibited by MiR-20a-5p Overexpression in MPP^+^-Treated HT22 Cells

**Figure 2:**
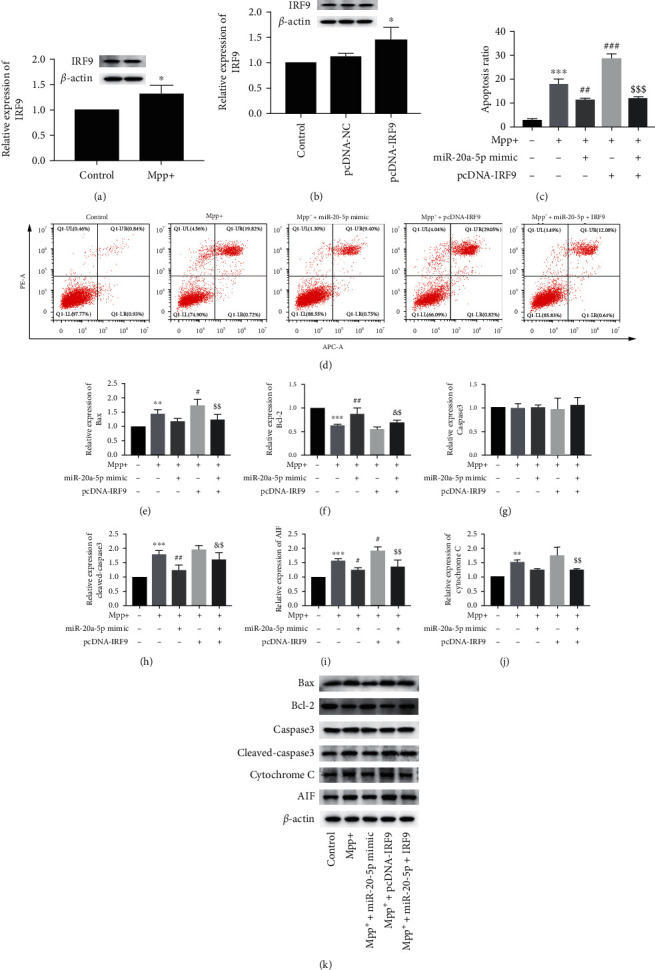
IRF9 reversed cell apoptosis inhibited by miR-20a-5p overexpression in MPP^+^-treated HT22 cells. HT22 cells were pretreated with miR-20a-5p mimic and/or pcDNA-IRF9 for 24 h and then treated with MPP^+^ (0.5 mM) for 24 h. (a and b) The protein level of IRF9 was tested by Western blot analysis. *β*-actin is a loading control.  ^*∗*^*P* < 0.05 vs. pcDNA-NC. (c and d) The cell apoptosis was assessed by Annexin V FITC/PI staining flow cytometry. (e–k) The expression of Bcl-2, Cleaved-Caspase 3, Bax, AIF, and cytochrome C was determined by Western blot analysis. *β*-actin is a loading control.  ^*∗∗*^*P* < 0.01 vs. control group,  ^*∗∗∗*^*P* < 0.001 vs. control group, ^#^*P* < 0.05 vs. MPP^+^-treated group, ^##^*P* < 0.01 vs. MPP^+^-treated group, ^&^*P* < 0.05 vs. MPP^+^ + miR-20a-5p mimic-cotreated group, ^$^*P* < 0.05 vs. MPP^+^ + pcDNA-IRF9-cotreated group, and ^$$^*P* < 0.01 vs. MPP^+^ + pcDNA-IRF9-cotreated group. Data are expressed as mean ± SD. The experiments were repeated six times.

As shown in Figure 2(a), compared with the control group, the IRF9 protein expression was increased in MPP^+^-treated HT22 cells. So, we synthesized an effective overexpression vector (pcDNA-IRF9) to discuss the effect of IRF9 on MPP^+^-induced HT22 cell injury (Figure 2(b)). First, our results showed that the overexpression of miR-20a-5p and IRF9 in HT22 cells inhibited and promoted cell apoptosis induced by MPP^+^ treatment, respectively (Figures 2(c) and 2(d)). Additionally, in comparison to the control, the protein expression of caspase 3 was unchanged, the protein expression of Bcl-2 was decreased, and the protein expression of cleaved-caspase 3, Bax, AIF, and cytochrome C significantly was increased in MPP^+^-treated groups (Figures 2(e)–2(k)). Of note, miR-20a-5p overexpression remarkably inhibited the cleaved-caspase 3 and AIF expression and promoted Bcl-2 expression. On the contrary, IRF9 overexpression dramatically activated Bax and AIF expression. Meanwhile, cotransfection of pcDNA-IRF9 and miR-20a-5p mimic reversed these changes (Figures 2(e)–2(k)).

### 3.4. IRF9 Offset Mitochondrial Dysfunction Attenuated by MiR-20a-5p Overexpression in MPP^+^-Treated HT22 Cells

**Figure 3:**
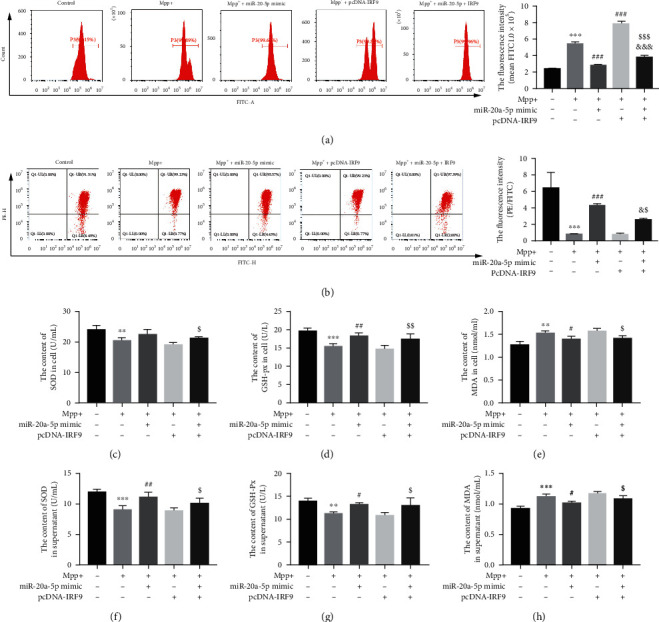
IRF9 offset mitochondrial dysfunction attenuated by miR-20a-5p overexpression in MPP^+^-treated HT22 cells. HT22 cells were pretreated with miR-20a-5p mimic and/or pcDNA-IRF9 for 24 h and then treated with MPP^+^ (0.5 mM) for 24 h. (a) Reactive oxygen species (ROS) activity was performed using DCFH-DA. (b) The mitochondrial membrane potential (MMP) was assayed by JC-1 fluorescent probe. The contents of SOD, GSH-XP, and MDA in cells (c–e) and supernatant (f–h) were tested by enzyme-linked immunosorbent assay (ELISA).  ^∗∗^*P* < 0.01 vs. control group,  ^∗∗∗^*P* < 0.001 vs. control group, ^#^*P* < 0.05 vs. MPP^+^-treated group, ^##^*P* < 0.01 vs. MPP^+^-treated group, ^###^*P* < 0.001 vs. MPP^+^-treated group, ^&^*P* < 0.05 vs. MPP^+^ + miR-20a-5p mimic-cotreated group, ^&&&^*P* < 0.001 vs. MPP^+^ + miR-20a-5p mimic-cotreated group, ^$^*P* < 0.05 vs. MPP^+^ + pcDNA-IRF9-cotreated group, ^$$^*P* < 0.01 vs. MPP^+^ + pcDNA-IRF9-cotreated group, and ^$$$^*P* < 0.001 vs. MPP^+^ + pcDNA-IRF9-cotreated group. Data are expressed as mean ± SD. The experiments were repeated six times.

Accumulating evidence suggests that mitochondrial dysfunction may lead to cell damage in the form of ROS production and MMP decreases. As shown in Figures 3(a) and 3(b), the ROS production was increased, while the MMP was significantly decreased in MPP^+^-treated HT22 cells. In addition, the mitochondrial ROS level was decreased and MMP level was increased in miR-20a-5p mimic-transfected HT22 cells. However, the transfection of pcDNA-IRF9 showed the opposite results (Figures 3(a) and 3(b)). In addition, cotransfection of pcDNA-IRF9 and miR-20a-5p mimic significantly restored the decrease of ROS production and the increase of the MMP level induced by miR-20a-5p mimic (Figures 3(a) and 3(b)). Meanwhile, the ROS production was decreased and the MMP level was increased in pcDNA-IRF9 and miR-20a-5p mimic cotransfected HT22 cells compared with the pcDNA-IRF9-transfected group (Figure 3(a)).

It is well known that the content of MDA reflects the degree of oxidative stress damage of cells. And, SOD and GSH-PX levels reflect the antioxidant capacity of cells. In our study, we found that the MDA content was increased, and GSH-Px and SOD content were decreased in MPP^+^-treated HT22 cells and supernatant (Figures 3(c)–3(h)). Conversely, miR-20a-5p mimic reversed these changes (Figures 3(c)–3(h)). Moreover, the MDA production and secretion were inhibited in pcDNA-IRF9 and miR-20a-5p mimic cotransfected HT22 cells compared with the pcDNA-IRF9-transfected group, and the GSH-Px and SOD content showed the opposite results (Figures 3(c)–3(h)).

### 3.5. MiR-20a-5p Overexpression Diminished Inflammatory Response in MPP^+^-Treated HT22 Cells Partially through IRF9/NF-*κ*B Axis

**Figure 4:**
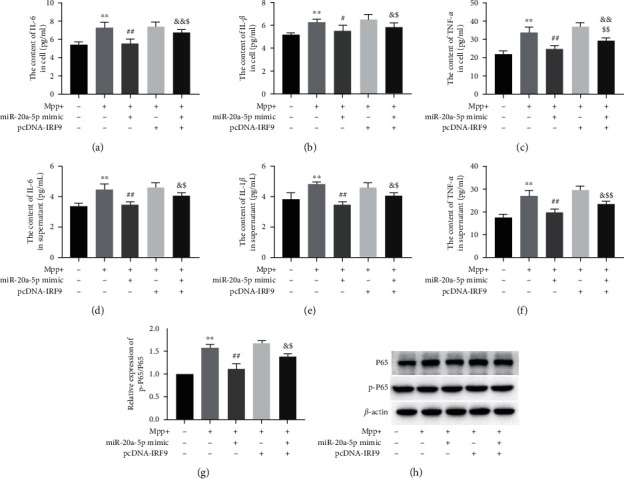
MiR-20a-5p overexpression diminished inflammatory response in MPP^+^-treated HT22 cells partially through IRF9/NF-*κ*B axis. HT22 cells were pretreated with miR-20a-5p mimic and/or pcDNA-IRF9 for 24 h and then treated with MPP^+^ (0.5 mM) for 24 h. The contents of IL-1*β*, IL-6, and TNF-*α* in cells (a–c) and supernatant (d–f) were tested by enzyme-linked immunosorbent assay (ELISA). (g and h) The p-P65 expression was determined by Western blot analysis. *β*-actin is a loading control.  ^*∗*^*P* < 0.05 vs. control group,  ^∗∗^*P* < 0.01 vs. control group,  ^∗∗∗^*P* < 0.001 vs. control group, ^#^*P* < 0.05 vs. MPP^+^-treated group, ^##^*P* < 0.01 vs. MPP^+^-treated group, ^&^*P* < 0.05 vs. MPP^+^ + miR-20a-5p mimic-cotreated group, ^&&^*P* < 0.01 vs. MPP^+^ + miR-20a-5p mimic-cotreated group, ^$^*P* < 0.05 vs. MPP^+^ + pcDNA-IRF9-cotreated group, and ^$$^*P* < 0.01 vs. MPP^+^ + pcDNA-IRF9-cotreated group. Data are expressed as mean ± SD. The experiments were repeated six times.

As shown in Figures 4(a)–4(f), the production and secretion of IL-1*β*, IL-6, and TNF-*α* were all enhanced after MPP^+^ treatment. In the case of MPP^+^-treated cells, transfection with miR-20a-5p mimic inhibited the production and secretion of IL-1*β*, IL-6, and TNF-*α*, which were significantly reversed by IRF9 overexpression. Additionally, pcDNA-IRF9 and miR-20a-5p mimic cotransfected significantly decreased the content of IL-1*β*, IL-6, and TNF-*α* in supernatant and secretion compared with the group transfected with pcDNA-TRF9 alone (Figures 4(a)–4(f)). As an important inflammatory response regulator, NF-*κ*B was reported to widely mediate neuroinflammation in PD [16]. Mechanically, miR-20a-5p overexpression decreased the p-P65 expression in MPP^+^-treated HT22 cells. Meanwhile, the decrease of p-P65 level induced by miR-20a-5p mimic was significantly blocked by IRF9 overexpression (Figures 4(g)–4(h)). These results suggested that IRF9 could reverse the inhibition effect of miR-20a-5p overexpression on inflammation of MPP^+^-treated HT22 cells; notably, this might only be a partial effect through NF-*κ*B activation based on the results shown in Figures 4(g)–4(h).

## 4. Discussion

It has been reported that MPP^+^ induces oxidative damage by selectively inhibiting the activity of mitochondrial respiratory chain complex I, blocking NADH oxidative phosphorylation system, and reducing ATP generation in dopaminergic neurons or cortical neurons [17, 18]. Therefore, MPP^+^ is currently used to establish in vitro models of PD. In the present study, significant decreases of HT22 cell viability were caused by MPP^+^ at concentrations of 0.5, 1, and 2.5 mM. We chose 0.5 mM MPP^+^ for subsequent experiments because the influences of miR-20a-5p or IRF9 might have been masked if severe MPP^+^ toxicity was induced.

Past studies indicated that oxidative stress and downstream neuroinflammation in the brain play key roles in contributing to neurodegeneration and neuronal death in PD [19]. Notably, the anti-inflammatory effect of miR-20a-5p has been widely recognized. MiR-20a-5p decreased allergic inflammation in HMC-1 mast cells by targeting HDAC4 [20]. MiR-20a-5p/TGFBR2 axis resulted in activation of TGF-*β* signaling pathway and regulated inflammation-driven liver fibrosis [21]. However, the effect of miR-20a-5p on cell mitochondrial dysfunction and oxidative damage has not been reported. In addition, there is increasing evidence proving that miR-20a-5p had an excellent role in neuronal cell proliferation and neural function maintenance. The study found that miR-20a-5p inhibited neuroblastoma proliferation and autophagy and promoted cell apoptosis through negative regulation of ATG7 [22]. Network-based transcriptome data analyses suggested that miR-20a-5p was an important regulatory molecule and might be a potential drug target for the AD [23]. Meanwhile, the expression of miR-20a-5p was significantly increased in rat hippocampus from 24 hours to 1 week after status epilepticus (SE) induced [24]. Emerging research showed that miR-20a-5p-RGMa-RhoA signaling pathway regulated axonal growth and neuronal branching in vitro and regulated epileptogenesis in vivo [25]. Our evidence further supported that miR-20a-5p attenuated MPP^+^-induced cytotoxicity in HT22 cells, including reduction in the proportion of apoptosis and weakening of inflammation and oxidative stress, suggesting that overexpression of miR-20a-5p had protective effect on MPP^+^-induced neuron death.

Previous research displayed that, following intracranial infection with virus, the expression of interferon regulatory factor 9 (IRF9) was enhanced in neurons [26], implying that IRF9 may be involved in the regulation of central nervous system. IRF9 is a member of a family of interferon regulatory factors and plays an important role in antivirus, immune response, cell growth regulation, and apoptosis. In particular, IRF9 is a member of the interferon (IFN)-stimulated gene factor 3 (ISGF3) complex, which consists of STAT1, STAT2, and IRF9. Under the stimulation of INF, IRF9 can bind to the promoter of the IFN-induced genes (ISGs) to enhance gene transcription and finally mediate the antiviral effect of INF [27]. During dextran sodium sulfate- (DSS-) induced colon inflammation and IFN-*γ*-treated macrophages, STAT1/IRF9 complex played a proinflammatory effect by regulating the transcription of CXCL10 gene [28]. Simultaneously, STAT1/IRF9 complex could bind tightly to the p65 subunit of NF-*κ*B and increased synthesis of IL-6 [29]. In our experiments, IRF9 reversed the improvement of miR-20a-5p overexpression on HT22 cell inflammation, oxidative stress, and apoptosis induced by MPP^+^. Furthermore, the decrease of p-P65 level induced by miR-20a-5p mimic was significantly blocked by IRF9 overexpression.

## 5. Conclusions

Together, we found that miR-20a-5p overexpression alleviated MPP^+^-induced inflammation and oxidative stress response in HT22 cells. Neuroprotective effect of miR-20a-5p was achieved in part by targeting IRF9/NF-*κ*B axis.

## Data Availability

The datasets used or analyzed during the current study are available from the corresponding author upon reasonable request.

## Additional Points

*Highlights*. MiR-20a-5p level was decreased in MPP^+^-induced HT22 cells. IRF9 was a target gene of miR-20a-5p. IRF9 hindered the improvement of miR-20a-5p overexpression on MPP^+^-induced neurotoxicity.
